# A Path to a Reduction in Micro and Nanoplastics Pollution

**DOI:** 10.3390/ijerph20085555

**Published:** 2023-04-18

**Authors:** Jay N. Meegoda, Mala C. Hettiarachchi

**Affiliations:** 1 Department of Civil and Environmental Engineering, New Jersey Institute of Technology, Newark, NJ 07102, USA; 2 Environmental Resources Group, Wixom, MI 48393, USA

**Keywords:** microplastics, nanoplastics, environment and human beings, wastewater, policy

## Abstract

Microplastics (MP) are plastic particles less than 5 mm in size. There are two categories of MP: primary and secondary. Primary or microscopic-sized MP are intentionally produced material. Fragmentation of large plastic debris through physical, chemical, and oxidative processes creates secondary MP, the most abundant type in the environment. Microplastic pollution has become a global environmental problem due to their abundance, poor biodegradability, toxicological properties, and negative impact on aquatic and terrestrial organisms including humans. Plastic debris enters the aquatic environment via direct dumping or uncontrolled land-based sources. While plastic debris slowly degrades into MP, wastewater and stormwater outlets discharge a large amount of MP directly into water bodies. Additionally, stormwater carries MP from sources such as tire wear, artificial turf, fertilizers, and land-applied biosolids. To protect the environment and human health, the entry of MP into the environment must be reduced or eliminated. Source control is one of the best methods available. The existing and growing abundance of MP in the environment requires the use of multiple strategies to combat pollution. These strategies include reducing the usage, public outreach to eliminate littering, reevaluation and use of new wastewater treatment and sludge disposal methods, regulations on macro and MP sources, and a wide implementation of appropriate stormwater management practices such as filtration, bioretention, and wetlands.

## 1. Introduction

The total global plastics production in 2021 was estimated to be 390.7 million metric tons with an annual increase of four percent [1]. The plastics production has soared since 1950. The versatility of plastics has accounted for this continued growth in production. A large fraction of plastics is produced for single-use purposes such as packaging. Additionally, more than 10,000 chemicals are used in manufacturing plastics. Out of these, at least 2000 substances would create human or environmental health impacts [2]. For example, bisphenol A (BPA) and phthalates, prevalent in plastic manufacturing, are associated with negative health impacts such as infertility, cardiovascular diseases, and cancer. As of 2015, approximately 6300 million metric tons (Mt) of plastic waste had been generated, around 9% of which was recycled, 12% was incinerated, and 79% was accumulated in landfills or in the natural environment [3]. If current production and waste management trends continue, roughly 12,000 Mt of plastic waste will be in landfills or in the natural environment by 2050. Plastic is becoming increasingly common in nature, due to their poor biodegradability, massive production rate, and poor waste management. The abundance of plastic waste in the environment has led to another problem—microplastic and nanoplastic pollution. Microplastics (MP) are small plastic pieces that are less than 5 mm (mm) in length. Nanoplastics (NP) are generally classified as plastics smaller than 1 micrometer (µm). However, the definition of NP is still evolving. Microplastic pollution has become a global environmental problem due to their abundance, poor biodegradability, toxicological properties, and negative effects on aquatic and terrestrial organisms including humans. 

MP can be made up of many different plastic types such as polyethylene terephthalate (PET), polyethylene (PE), polypropylene (PP), polystyrene (PS), and polyvinyl chloride (PVC). Each plastic type creates unique environmental problems. For example, PE produces methane and ethylene when exposed to sunlight, contributing to climate change problems [4]. MP could contain toxic chemicals that are deliberately added into the plastic to give them enhanced properties such as better strength, better flexibility, stiffness, and various other desirable properties. In the aquatic environment, MP could adsorb toxic chemicals, bacteria, and pathogens from their surroundings [5,6,7,8,9,10,11,12]. In terrestrial environments, MP can affect soil fertility and soil organisms [13]. For example, they can affect earthworm development and mortality.

This manuscript aims to discuss the best way to mitigate the MP and NP pollution.

## 2. Micro and Nanoplastic Types

MP and NP can be of any type of plastic polymer and are categorized into two types: primary and secondary. These types differ in origin. MP come in many different shapes, such as fragments, fibers, pellets, foams, films, and more. Both MP and NP can also be of any color.

### 2.1. Primary MP and NP

Plastics that are manufactured to be of a microscopic size for commercial use are defined as primary MP. These plastics are typically used in facial cleansers and cosmetics, while their use in medicine as vectors for drugs is also reported [14]. Due to recent regulations on microbeads, microplastic “scrubbers”, used in hand cleansers and facial scrubs, have been replaced with traditionally used natural ingredients. Since the patenting of microplastic scrubbers within cosmetics in the 1980s, the use of exfoliating cleansers containing plastics has risen dramatically [15]. Typically marketed as “microbeads” or “microexfoliates”, these plastics can vary in shape, size, and composition depending upon the product. For example, Gregory [16] reported the presence of PE and PP granules (<5 mm) and PS spheres (<2 mm) in one cosmetic product. More recently, Fendall and Sewell [17] reported an abundance of irregularly shaped MP, typically <0.5 mm in diameter with a mode size < 0.1 mm, in other cosmetic products.

### 2.2. Secondary MP and NP

Fragmentation of large plastic debris such as plastic litter, fiber from synthetic textiles, and tires through physical, chemical, and oxidative processes creates secondary MP [18,19]. Weathering is the primary process for plastic degradation [20,21]. Additionally known as photofragmentation, ultraviolet (UV) radiation from the sun oxidizes the plastic polymer, resulting in chain scission [22]. The photofragmentation causes the material to become brittle until mechanical failure. The loss of structural integrity makes these plastics susceptible to fragment into MP in the presence of abrasion, wave action, or turbulence. Galgani [23] reported that the smallest microparticle detected in the oceans was 1.6 μm. However, when the external environment changes, such as in the low-energy marine environment of the benthic zone, the degradation rate of MP slows down significantly [24]. In addition, Dawson [25] reported conversion of MP into NP through digestive fragmentation.

## 3. Sources of MP and NP

There are multiple sources of MP including paint, pellets, synthetic textile fibers, and tire dust. MP tend to accumulate in the ocean or aquatic environment through runoff, river transport, or direct discharge [26]. MP also leach into the groundwater or remain in the soil. 

Wastewater treatment plants (WWTPs) are one of the primary routes of entry for MP into the environment. The wastewater received by WWTPs from residential, commercial, and industrial sources contains MP due to the use of various consumer products that contain or emit MP. In general, WWTPs treat wastewater in three stages: primary, secondary, and tertiary. Primary treatments physically remove insoluble solids such as MP via a variety of methods including screening, grit removal, and primary settling. Secondary treatment includes biological treatments and secondary settling. The two processes could partition as much as 88% MP into the wastewater sludge [27,28,29]. Tertiary treatment is where the advanced wastewater treatment processes such as filtration are employed. Overall, the primary, secondary, and tertiary treatment could remove over 90% of MP from water. However, removal of extremely small MP particles and NP require more advanced treatment methods such as nanofiltration (NF) or reverse osmosis (RO). As concern about MP pollution grows, the potential for wastewater technologies to capture particles before they reach surface waters has begun to attract attention [28]. 

Landfills are also a source of MP. Carr [30] found elevated concentrations of MP in proximity to landfills, indicating them as a potential source. Typically, landfill leachate is disposed at WWTPs. Landfill leachate could contain microplastics as sanitary landfills are the final disposal sites of municipal waste containing plastics. The plastic waste in landfills degrades into MP over time, some of which enters the leachate. 

MP captured to sewage sludge is another significant source. The MP captured into the sewage sludge is generally land applied as biosolids, landfilled, or incinerated. When the biosolid or sewage sludge is disposed in sanitary landfills, the MP could return back to the WWTPs via leachate. Additionally, the size of MP could be further reduced within the landfills. The MP contained in land-applied biosolids could contaminate terrestrial ecosystems and also re-enter the aquatic environment via stormwater runoff [31]. While the fate and transport of MP could depend on the concentrations, as well as the relative proportions of particle types, they create a damaging impact in the receiving environment [32,33,34,35,36,37,38]. 

Food waste is another important route for MP to enter aquatic environments and the human body. The plastic contamination in pre-cooked instant rice could be four times greater than in uncooked rice [39]. The plastic packing could produce MP when immersed in hot water, microwaving, or refrigeration. MP from Styrofoam (a commonly used disposable food container) are released after being subject to high temperatures [40,41]. Furthermore, unsorted plastic waste such as plastic bags, disposable cutlery, and packaging inevitably mixes with food waste during disposal. Many countries and municipalities allow for food waste to be collected in plastic bags. This mixed waste is difficult to properly recycle and is disposed in landfills where the plastics degrade into MP over time. In some instances, plastic bags are used to store and transport food waste that is composted. This composted food waste with shredded plastic is used as a fertilizer. During composting and subsequent farming, these plastics are further degraded. The MP in the soil is then taken up by plants leading to human or animal consumption [42].

Other significant sources of microplastics include synthetic rubber fragments from the wear and tear of tires, artificial sport turfs, and encapsulation of controlled-release fertilizers. Macroplastics such as single use plastic mulch and plastic litter are also sources of MP as they degrade into MP over time. MP from these sources could reach aquatic environment via stormwater runoff. Therefore, stormwater is also a significant pathway of microplastics to the environment.

## 4. Plastic Recycling

Recycling can reduce the amounts of plastics that enter the environment or landfills. Those are two final destinations for more than three quarters of non-degradable plastic waste [3]. The recycling of plastic waste in the United States has decreased in recent years from the rate of 9.0% of the total plastic waste material recycled in 2015 to 8.4% of the total plastic waste recycled in 2017 [43]. The weight burned for energy recovery in 2017 was 5.6 million tons, up 4.9% from 2015. The weight landfilled was 26.8 million tons, up 3% over 2015. The 8.4% recycling rate covers plastics used in both durable and nondurable products. The waste data from USEPA [43] showed that the total percentage of material landfilled is over 75% of the total amount of plastic waste generated, indicating the need of proper waste management practices. 

To promote recycling, appropriate regulations on plastic waste management are required. For example, Germany recycled more than 99.6% of plastic packaging waste in 2019 due to the packaging ordinance. California’s State Bill 54 requires all packaging in the state to be recyclable or compostable by 2032, cutting plastic packaging by 25% in 10 years and requiring 65% of all single-use plastic packaging to be recycled in the same timeframe.

## 5. Human Health Impacts and Source Reduction

MPs enter the human body via inhalation, contact, and consumption [44]. Most MP are consumed via drinking water and food. Since most water treatment plants are unable to remove NP and most of the finer MP, they are found in drinking water, a significant exposure pathway [45,46,47,48,49,50]. Zuccarello [51] showed MP (<10 μm) associated to plastic water bottles, another source of drinking water. Thiele [52] showed that fishmeal is a source of MP to the environment and human. Li [53] found that MP are indeed contaminating edible plants. 

The consumed NP and MP via water and food could create adverse health effects such as impairments in oxidative and inflammatory intestinal balance, problems in immunotoxicity, and gut microbiota disruption [54,55,56]. The consumed NP and MP could also translocate into organs, blood, or even breastmilk [57,58,59,60,61,62,63,64]. 

Removal of MP from the environment is challenging. Source reduction is one of the best methods available to protect the environment and human health. This includes reducing the usage, and public outreach to eliminate littering. In addition, regulations on single-use packaging material could reduce the plastic pollution as most plastic items found in the aquatic environment are created for single use purposes. Elimination or minimization of the use of proven or potentially hazardous chemicals in manufacturing of consumer products such as food containers could help reduce the negative health impacts. Use of less plastic in packaging could also help reduce the pollution. Elimination of the overuse of compost and fertilizers could reduce soil contamination with MP [65]. In addition, source reduction approaches should be developed for all major MP sources such as paints, synthetic textile fibers, and tire wear dust. 

## 6. Strategies to Reduce the MP Pollution

This section describes some of the strategies to reduce MP pollution. While novel technologies such as chemical recycling, a process in which the waste materials are converted into their basic components, are under development, these strategies could help reduce further contaminating the environment with MP.

### 6.1. Remediation of Stormwater

Bioretention cells, a type of treatment for stormwater, are effective in removing MP from urban stormwater [66]. Bioretention cells are depressions in the ground where stormwater runoff is collected and treated. Smyth [66] showed that MP with a size of 106–5000 μm produced an 84% median decrease. 

### 6.2. Remediation Technologies for Water/Wastewater

A large portion of MP are removed by the WWTPs. However, appropriate wastewater sludge management techniques must be implemented at WWTPs to stop the reentry of MP into the environment. For MP rich biosolids, bioremediation could be used as a treatment method. Bioremediation is the use of microorganisms to break down the MP via hydrolysis [67]. Some microorganisms (such as bacteria or fungi) can break down plastics by secreting enzymes that perform hydrolysis on MP [65]. 

#### 6.2.1. Physical Treatment 

Physical treatment processes such as filtration, coagulation, flocculation, and sedimentation could be utilized in wastewater treatment to remove MP [68,69,70,71]. Removal of MP from water through coagulation, flocculation, and sedimentation (CFS), widely used wastewater treatment methods, seem to be effective. The removal efficiency is dependent on several different factors including coagulant type and dose, characteristics of water sample, and characteristics of MP. Shahi [72] reported that MP of 30–100 μm were completely removed via CFS. Several other studies have investigated the impact of coagulant type and dose on the removal efficiency of MP. Generally, polyaluminium chloride (PAC), a widely used coagulant, seems to be the most effective in removal of MP from water samples. Na [73] tested both aluminum chloride and iron chloride as coagulants for the removal of MP sized 10–90 μm. Rajala [74] investigated the effectiveness of ferric chloride, PAC and polyamine as coagulants for removing MP. The highest removal efficiency of 99.4% was observed for MP sized 1 micrometer with ferric chloride as the coagulant [74]. Zhang [71] investigated the effect of PAC and polyacrylamide (PAM) dose on the removal of NP sized 50–1000 nm. Zhang [70] investigated the effectiveness of magnetic magnesium hydroxide as a coagulant for the removal of MP from wastewater. Additionally, Zhang [70] tested the effectiveness of Polyacrylamides (PAM) as a flocculant to aid in the process. Shi [75] showed that MP can be removed from aqueous solutions by magnetized nano-Fe_3_O_4_ to magnetize MP and remove them from the water. A similar method can also be used with aluminum and iron-based salts, which have chemical formulas of AlCl_3_·6H_2_O and FeCl_3_·6H_2_O, respectively. According to Singh [76], this process showed high removal efficiency for smaller-sized MP. Electrocoagulation is another physical process available for removing MP from wastewater. In this process, a positively charged ion (usually a Fe^2+^ ion or an Al^3+^ ion) and a negatively charged ion are used as coagulants to form sludge. The MP-containing sludge can be removed via settling. Another potential solution is the utilization of biochar, charcoal produced in the absence of oxygen. When utilized in filtration processes, biochar is extremely effective in removing MP. According to Singh [76], biochar removed >95% of MP > 10 μm. Zirconium metal foams also show great efficacy, stopping ~95% of MP via entanglement within the metal foam. Photocatalytic titanium dioxide micromotors (miniature, self-propelled devices) have also emerged as a viable solution for removing MP. 

#### 6.2.2. Chemical Treatment

Advanced oxidation processes (AOPs) can remove MP when implemented as a tertiary treatment method. Several authors have proposed various AOPs to degrade MP and NP [77,78,79,80,81,82,83,84,85]. These are processes that use hydroxyl-containing free radicals to cause oxidation reactions with a target molecule to be removed. This is often deployed during tertiary treatment in WWTPs. Numerous tests of AOPs show good efficacy in removal of MP, and it is seen as a promising, novel treatment that is also eco-friendly. There are numerous AOPs including ozonation, utilizing ozone, or utilizing Fenton’s reagent. Luo [82] showed that AOPs were effective in breaking down MP additives such as pigments, and that they were destroyed at a higher rate than additives leached out into the environment. AOPs help accelerate the aging and degradation of MP, as well as removal. The number of commercialized AOP applications around the world is increasing, especially in the United States and Europe. However, AOPs are expensive, as for them to operate, a constant source of hydroxyl radicals is required. 

#### 6.2.3. Biological Treatment 

The biological treatment which includes biodegradation and bioremediation is an effective way to degrade MP and convert them into environmentally friendly carbon compounds Actinomycetes, algae, bacteria, fungi, and their enzymes enhanced the degradation of synthetic plastics [12,67,86,87,88,89,90,91]. The application of different microorganisms as a consortium would lead to greater efficiency in plastic degradation due to the synergism between the microbes and their enzymes [90]. 

#### 6.2.4. Remediation Challenges

The MP removal efficiency depends on the water characteristics such the pH. Zhang [72] found that the optimal pH for removal was 8, using a PAC dose of 400 mg/L and PAM dose of 20 mg/L. Rajala [74] found that at a pH of 6.5, higher doses of the coagulants were needed for more efficient removal of MP. Conversely, at a pH of 7.3, lower doses of the coagulants were required for the removal of MP, wherein higher doses led to a decline in the removal efficiency. Xue [92] investigated the effect of the chemical characteristics of water on the removal efficiency of MP and found that the removal efficiency of MP in Lake Erie water was noticeably higher than that in Grand River water. Xue [92] reasoned that the reason for this might be the higher turbidity of Lake Erie, suggesting that the initial turbidity of the influent significantly affects the removal efficiency of MP. The size of MP impacts the removal efficiency. The removal efficiency of particles smaller than 500 μm was very low in operating WWTPs [93]. However, in lab-scale studies, the removal efficiency of particles sized 30–100 MP was found to be more significant. Na [73] found that the removal efficiency of MP sized 20–90 μm was 77.4–95.3%, while that of particles sized 10 μm was only 33–41.4%. In other studies, the removal efficiency of particles smaller than 10 μm was also found to be significant (>90%). Shahi [72] found that the elongated MP were removed more efficiently than spherical MP, and that MP with rougher surfaces were removed more efficiently than MP with smoother surfaces. Challenges associated with quantification of MP hinders the selection of appropriate technologies for remediating various sources of MP [94,95].

As stated before, WWTPs are one of the primary routes of MP into the environment. Existing studies seldom investigate the removal efficiency of NP, particularly at existing WWTPs, since most particles detected with current detecting technologies exceed 1 micrometer [95]. This suggests that the current detection methods must be enhanced in order to better study the effect of size on the removal efficiency [96,97]. Bio- and photodegradation are more appropriate for complete degradation and mineralization, but they require a comparatively long time to achieve adequate remediation [98]. However, there are not widely accepted and commercially successful remediation technologies to destroy MP and NP.

### 6.3. Other Challenges

There are several challenges facing MP research. Firstly, there are few protocols for detection and measuring the concentration of MP and especially NP in a given sample. Due to their small size, MP are simply difficult to retain and can easily escape filtration. Additionally, more understanding is needed on the fate of MP in soil and living matter, and how they affect other nutrients or pollutants in their respective environments. Due to the additives and plasticizers used in producing plastics, each plastic type may be slightly different from one another [75]. This means that not only microplastic toxicity [99] but also adherent toxicity are concerns. The majority of MP found in aquatic environment are secondary MP. Therefore, a potential way to reduce secondary microplastic pollution would be the reduction in the overuse of plastics [100,101,102,103,104,105,106,107]. 

Plastic is an important engineering material with a multitude of benefits. The usage of plastics created lightweight cars, reducing greenhouse gases. Plastics are widely used because they are lightweight, durable, flexible, moldable to one’s needs, and, most importantly, inexpensive. Therefore, it would be hard to reduce the production of such a beneficial material unless a better alternative is discovered. Therefore, the usage of plastics can be reduced by using biodegradable materials. The use of known toxic substances can be banned in creating consumer products such as food containers to protect human health. Public education of the effect of plastic in schools and institutions of learning is a good way to raise awareness and is a long-term potential solution. Additionally, awareness also goes hand in hand with education, and it has been shown that people who are aware of MP and their effects are more likely to refuse microbead (a shape of microplastic) products [108]. Proper waste disposal and management is also a way to prevent plastic from entering the environment and deteriorating into MP. For example, in Taiwan, waste management policies such as bans of plastic bags and tableware and the sorting of waste to ensure proper disposal were effective in reducing waste disposal rate (from 0.9 kg per capita to 0.48) [12]. However, proper waste management systems and centers are costly and hard to implement; thus, a more viable solutions for communities are needed. Another option is to attempt to hold global companies more accountable and make them remove unnecessary elements of packaging that may contain plastics. 

### 6.4. Current Legislative Approaches to Limit MP Pollution

Global efforts have been put into place to reduce the amount of MP and NP in the environment. The United Nations Sustainable Development Goal 14 targets significant reduction in marine pollution by 2025. Additional measures of microplastic legislation have also been passed in several developed nations. In 2015, the United States Congress amended the Federal Food, Drug, and Cosmetic Act with the Microbead-Free Waters Act of 2015 [108]. This bill prohibits the manufacture/packaging/distribution of rinse-off cosmetics that contain plastic microbeads. A rinse-off cosmetic, as the name implies, is any cosmetic product intended to be removed after use (i.e., shampoo, shaving cream, toothpaste). In this scenario, the law describes a “plastic microbead” as any plastic particle of less than 5 mm in size, fitting with the current definition of MP. This law is meant to target the production of primary MP intentionally added to rinse-off cosmetic products [109]. However, this ban is not included regarding other primary microplastic-containing products. The European Union has stated in the past that they will address the release of MP into the environment [109]. In addition, the EU aims to develop a system of labeling, standardization, and regulation on unintentionally released MP, develop methods for measuring the unintentional release of MP, and gain additional scientific knowledge on the effects of MP. The European Chemicals Agency (ECHA), an agency of the European Union, has proposed restrictions on production of MP. The European Commission, the European Union’s governing body, has said that they will reflect and assess on the restriction “soon”, assumably sometime in 2022. These are considered “first steps” in the EU’s microplastic initiative. Although there is no current comprehensive law governing MP in the EU, there are currently several specific laws that cover partial objectives. The EU currently has many laws on macroplastics, and also many laws concerning the production of MP and their release, both directly and indirectly. These include laws on wastewater treatment, sewage, and regulations on air quality and tires. Canada is currently in the process of creating laws and legislation to address MP. The UKWIR-recommended intervention control for wastewater industry [110] includes changes to labeling, extended producer responsibility, bans, grants and subsidies for reusables, and public infrastructure changes such as providing waste bins for disposal. In 2020, Canada proposed a new plastic waste management system that would, among other things, reduce the use of microbeads in personal care products, similar to the US legislation [108], as well as address other sources of MP [111]. It is a comprehensive management system, with an ultimate goal to reduce the amount of macro/MP that are discharged to the environment [111]. Canada surveyed people for this proposed management system to gain feedback from the public on the specificity and details of the management system. These feedback sessions ended in 2021. Canada, additionally, prohibited the manufacture and import of all toiletries with microbeads in them back in 2018, with a complete ban effective in 2019. In Australia, the Australian Institute of Marine Science is collecting data and learning more about MP for the Australian Government to then use. Australia has launched their National Plastics Plan 2021, which outlines and details a plan to phase out plastics in the Australian economy [112]. It plans to phase out problematic plastics, make oceans and beaches free of plastic, create legislation to ensure waste responsibility, invest in recycling capability, invest in research, and continue to support recycling. In 2020, Australia phased out microbeads in rinse-off cosmetics and PCPs. As the humanity move further and further into the future, the Australian government has even further goals, such as regulations on plastic waste and phasing out PVC plastic labels. In response to concerns about the impact of plastic and microplastic pollution, public engagement and political commitment has increased and more than 60 countries have already taxed or banned single-use plastics or plastic bags [113,114].

### 6.5. Path Forward-Public Outreach

**Figure 1:**
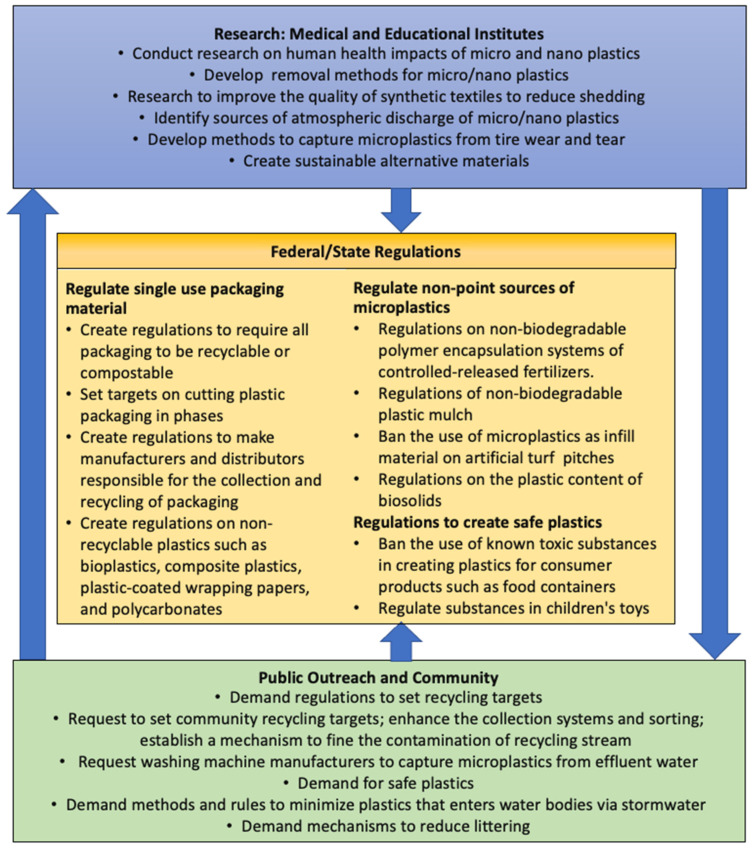
Action plan for reducing the MP and NP pollution.

Humanity currently faces two major challenges with respect to emerging environmental pollutants: per- and polyfluorinated substances (PFAS) and MP being absorbed by and retained in the human body, causing many negative health issues. For PFAS, there are substantial research funds dedicated to detection, monitoring, and destruction. Fortunately, there are various technologies available to destroy PFAS [115,116]. However, it is not the same for MPs. There are limited intensions to regulate and control the use of plastics and MPs. As outlined in Section 6.2, widely accepted and commercially successful remediation technologies to destroy MP and NP are not available. Hence, general public constantly ingest NP and MP via food and water. Drinking water becomes an exposure pathway of MP and NP for the communities that do not have access to advanced drinking water treatment systems such as reverse osmosis. Additionally, NP and MP enter the human body daily via food. The current public awareness of the negative health impacts of MP is unsatisfactory or non-existent. Hence, public education of the impact of MP on humans and environment is needed, urgently. Public awareness would create the urge to request new policies and regulation to mitigate MP and NP exposure. Implementation of these new policies requires the research community to perform additional research to further scientific knowledge and technology. Overall, the mitigation of MP and NP requires the involvement of the general public, regulators, educators, and medical professionals. Figure 1 provides an overview of the actions that could dramatically reduce the MP and NP entering the environment and human body.

## 7. Summary and Conclusions

MP are a growing concern due to their widespread presence in the environment and their negative effects on human health. Due to improper disposal and ineffective recycling, MP and NP are continuously generated and dispersed in the ecosystem. Recent research shows that humans regularly consume MPs and NPs via water and food. MP could be retained in the dietary track impeding the proper function of gut bacteria. The finer NPs permeate into the blood and other tissues and become accumulated in different parts of the human body. Hence, control of MP and NP are a major challenge for humanity. Hence, widely accepted removal technology for MPs is needed. Policies must be created to limit the production, consumption, and disposal of plastics. 

Understanding the sources of MP and development of treatment methods are crucial for addressing the MP issue. Additionally, information on health impact of plastics should be widely available to the general public to increase awareness and facilitate new policies to limit MP in the environment. While research on MP is increasing, more studies are needed in the following areas:

1. Standard detection methodologies;
2. The effects of chemical and particle toxicity on human health;
3. Quantification of MP sources such WWTPs and landfill leachate;
4. An accurate assessment of the MP in food;
5. Fate and transport of MP into crops;
6. Development of technologies and strategies for removing and possibly destroying MP;
7. New policies and regulations to reduce the usage of plastics and create safe plastics;
8. Public outreach is needed to demand new policies and regulations to reduce the usage of plastics and create safe plastics; and
9. Development of circular systems such as chemical recycling.
